# CEO Tenure, CEO Compensation, Corporate Social and Environmental Performance in China: The Moderating Role of Coastal and Non-coastal Areas

**DOI:** 10.3389/fpsyg.2020.574062

**Published:** 2021-01-22

**Authors:** Talat Mehmood Khan, Gang Bai, Zeeshan Fareed, Shakir Quresh, Zameer Khalid, Waheed Ahmed Khan

**Affiliations:** ^1^School of Finance, Southwestern University of Finance and Economics, Chengdu, China; ^2^School of Business, Huzhou University, Huzhou, China; ^3^School of Economics and Management, Xinjiang University, Urumqi, China; ^4^Department of Computer Science, COMSATS University Islamabad, Wah Cantt, Pakistan

**Keywords:** coastal areas, CEO compensation, CS&EP, career-concerns, stakeholder

## Abstract

This study uncovers a new finding on the impact of CEO tenure on corporate social and environmental performance (CS&EP) in coastal and non-coastal areas of China using fixed-effect panel data regression models. The Two-Stage Least Squares instrumental panel regression is used to validate the veracity of the empirical results. To this end, we extract data from all non-financial Chinese listed firms for the period of 2009 to 2015. By applying the multivariant framework, the findings of the study exhibit a negative and significant effect of CEO tenure on CS&EP. Moreover, this study shows that firms with head offices in coastal areas of China tend to weaken the negative impact of CEO tenure on CS&EP, indicating that CS&EP is more focused in coastal areas of China than non-coastal ones. The findings suggest that the increase in CEOs’ CS&EP in the early years of their service tenure tends to increase their compensation packages. This study is useful for policymakers to link CS&EP with firm economic practices to attain sustainable development objectives.

## Introduction

The extant literature signifies that social activities are performed for investment and signaling purposes (Su et al., 2016). In addition, stakeholder theory indicates that social projects are most likely to be considered as a stable investment to satisfy stakeholder expectations and consequently enhance firm performance (Wood, 1991; Price and Sun, 2017; Hu et al., 2018). In keeping with this argument, it is logical to anticipate that CEOs are more willing to initiate social projects to enhance firm performance. Accordingly, CEOs are more focused on organizing firm earnings during their initial tenures when boards of directors are keener on evaluating their capabilities regarding firm performance (Ali and Zhang, 2015). Moreover, due to the growing recognition of social activities at the firm level, executives are more likely to address social activities to improve their performance evaluations (Callan and Thomas, 2011; Hong et al., 2016; Liu and Zhang, 2017). Therefore, it is necessary to explore the differentiating role of CEO tenure on corporate social and environmental performance (CS&EP).

Coastal areas all around the world are attractive zones for both businesses and residences due to their abundant natural resources. Approximately 50% of the world’s population resides in coastal areas, which represents about 10% of the earth’s surface (Thia-Eng, 1993). Due to excessive population growth, pollution, over-exploitation of resources, and multiple resource-use conflicts, social development and environmental issues in coastal zones should be promoted and addressed through corporate social responsibility (Henceforth, CSR). China’s coastal areas cover around 18,000 km and extend across temperate, tropical, and subtropical zones (Cao and Wong, 2007; Qin et al., 2008). In China, about 60% of the population lives in the 12 coastal provinces. Moreover, around 70% of the biggest Chinese cities are situated in coastal areas and contribute to more than 55% of the country’s gross domestic product (Wang, 1992). The rapid urbanization, economic development and infrastructure development of China’s coastal areas increase their environmental deterioration and lead to ecosystem decline (Xue et al., 2004; Wu et al., 2012). The State Oceanic Authority is the only authority administering China’s coastal zones. The terrestrial land in the coastal areas does not fall under the State Oceanic Authority’s jurisdiction (Chen and Pearson, 2015). Therefore, the State Council of the People’s Republic of China (PRC) has implemented several laws to protect China’s coastal regions and enhance its social and environmental activities, such as the Law on the Territorial Sea and the Contiguous Zone (State Council of the PRC, 1992), the Law of the Exclusive Economic Zone and Continental Shelf (State Council of the PRC, 1998), and the Law on the Use and Management of Sea Areas (State Council of the PRC, 2001). In addition, the Chinese government has implemented integrated coastal zone management (ICZM), which is an internationally approved management tactic to address conflicts between economic development, social responsibility, and environmental protection to attain more sustainable coastal zone development (Chen and Pearson, 2015). The PRC’s ICZM project in Xiamen started in the mid-1990s and is considered the best example of ICZM execution (Hong and Xue, 2006). The ICZM project has now entered its second phase by accomplishing its required goals and receiving a positive response from the international community.

Approximately 70% of China’s biggest cities are situated in coastal regions, and contribute significantly to the country’s GDP (Wang, 1992). Therefore, the Chinese government is more focused on CS&EP projects in coastal zones. The role of firms in assisting and promoting social projects is much encouraged by the government and public sectors (Elkington, 1994; Laszlo and Cescau, 2017). Furthermore, multinational companies, like Nike, Carrefour, and GE, have urged CSR evaluations of their Chinese supplier firms (Ying et al., 2006). Approximately 8,000 Chinese firms situated in coastal zones have faced such factory evaluations (Ying et al., 2006). Hence, one primary task of Chinese firms is to upgrade their socially responsible competitiveness in the international market. All the pressure from the government, labor trade barriers, and green trade barriers has pushed Chinese firms to be more focused on CS&EP in coastal zones.

This paper extends the previous line of research by providing evidence of how the impact of CEO tenure on CS&EP varies in the coastal and non-coastal areas of China. Previous literature has shown a positive association between the CEO and the firm’s social performance (Li et al., 2020; Sarfraz et al., 2020b). Therefore, the variety of CEOs’ personality attributes and performance contracts provides a robust explanation for the dissimilarities of an organization’s CSR performance (Hambrick and Mason, 1984; Karim et al., 2018; Sarfraz et al., 2020b). CEOs have a strong association with a firm’s operational tasks. In most CEOs’ early service tenure, monitoring authorities, including government and a firm’s board of directors, are indecisive regarding the CEO’s capabilities. This leads to career concerns (Holmstrom, 1982; Gibbons and Murphy, 1992). In a wider prospect of cognitive development through moral socialization of CEOs, their ethical motivation varies across their life span (Kohlberg, 1969; Walker and Frimer, 2015). Therefore, executives demonstrating superior execution in their early service tenure ultimately reap greater rewards in their later service period in the form of self-governance, significant future remuneration, and service extensions (Hermalin and Weisbach, 1998). Due to the positive association between social practices and firm performance (Friede et al., 2015), CEOs in their early tenure are more likely to involve themselves in social practices and earnings overstatement to alleviate career concerns (Ali and Zhang, 2015). Therefore, we develop an argument that the increasing trend of CEO tenure tends to decrease the firm’s CS&EP. This is because CEOs address more CS&EP in the initial period of their service tenure to decrease career concerns, as social projects are considered a stable investment for extended service period (Palmer, 2012; Xiong et al., 2016). In the initial service period, CEOs are most likely to invest in social and environmental projects to reap rewards in their later years of service. To align these arguments, we propose an inverse association between CEO tenure and CS&EP. In the same vein, excessive government pressure, labor trade, and green trade barriers have pushed Chinese firms to be more focused on social activities in coastal areas (Ying et al., 2006). Therefore, we hypothesize that the inverse impact of CEO tenure on CS&EP is weaker in the coastal areas of China.

However, another interesting question remains to be answered: Does the CEO’s early service tenure engagement in social and environmental activities increase or decrease their compensation package in the Chinese context? We further extend our line of research to uncover the impact of CS&EP on CEOs’ total compensation. Conventional wisdom proposes that firms should accommodate CEOs’ desires to pursue social activities to raise the firm’s performance. Following corporate governance and the Global Reporting Initiative, the total compensation of top executives should be aligned with their social practices. For example, in 2013, the combined report of the Sustainable Investment Institutes and the Investor Responsibility Research Center indicated that 43% of Fortune 500 companies related top executive remuneration to their social activities^1^. However, the existing literature on the relationship between CEO compensation and social practices is inconclusive. On the one side, contradictory to conventional wisdom, researchers have observed that there is an inverse association between social activities and CEO compensation (Coombs and Gilley, 2005; Russo and Harrison, 2005), while on the other side, Berrone and Gomez-mejia (2009) have argued that environmental performance enhances CEOs’ compensation.

During a CEO’s early service tenure, the board of directors is more concerned with the CEO’s performance, which ultimately increases the CEO’s career-related concerns. Therefore, CEOs are more likely to address social practices to avoid the risk of dismissal and loss of pay (Oh et al., 2014). Extant research has shown that an increase in a firm’s social practices may increase its shareholders’ wealth (Fernández-Guadaño and Sarria-Pedroza, 2018). In the context of the present research, we propose that an increase in a CEO’s social and environmental practices is associated with an increase in shareholder wealth. The CEO’s CS&EP is positively correlated with their compensation package due to their social engagements (value-added hypothesis). Taken together, we propose that a CEO’s early-tenure CS&EP has a positive impact on their total compensation.

This study contributes significantly to the existing literature. To the best of our knowledge, this study is the first to comprehensively discern the link between CS&EP and CEO tenure in the coastal and non-coastal areas of China. Secondly, this study finds evidence that CEOs’ commitment to social and environmental goals is stronger in their initial service period than in their later service years. Third, we find that CEOs’ initial tenure investment in CS&EP could increase their total compensation. The rest of the study is arranged as follows. In section “Related Literature and hypothesis development,” we review the literature and present the hypothesis development. Section “Research Design” provides the model specification and sample size. Section “Main Results and Analyses” explains the results of the study and offers concluding remarks.

## Related Literature and Hypothesis Development

### Signaling Theory Effect

Signaling theory is based upon the assumption of information asymmetry and argues that a firm’s financial decisions work as a signal directed by the firm’s management to the stakeholders to address these asymmetries. In the present study, we expect CEO tenure to have an inverse impact on CS&EP for two reasons. First, in the initial service tenure, executives have more opportunities to engage in CS&EP to signal their ability to mitigate career-related concerns. In the early service period, the market is not confident in the newly hired CEO’s abilities (Holmstrom, 1982; Gibbons and Murphy, 1992). The CEO’s skills could be assessed based on financial and non-financial achievements (Chiu and Sharfman, 2016). Their unsatisfactory performance results in dismissal, low pay, and fewer perks (Deckop et al., 2006; Chiu and Sharfman, 2016). In the initial service period, CEOs are more interested in signaling their capabilities to reduce career-related concerns (Fama, 1980; Hermalin and Weisbach, 1998), while monitoring authorities consider financial and non-financial achievements as the benchmarks with which to assess their performance (Ali and Zhang, 2015; Hong et al., 2016). Moreover, Friede et al. (2015) recently reviewed the literature, finding that, in 2,200 studies, there was a positive association between a firm’s social and financial performance. Social performance is also an important indicator highlighting the capabilities of CEOs to reduce career risks, rather than conflicting with firm earnings (Ali and Zhang, 2015). Therefore, we propose that in the initial tenure of service, executives are more motivated to engage in social activities to alleviate career risks. Since social performance has been shown to be an important measure with which to assess CEO performance (Benabou and Tirole, 2010; Borghesi et al., 2014; Lys et al., 2015; Hong et al., 2016), we link it with CEOs’ career-related concerns.

Secondly, we postulate that an adverse effect of CEO tenure on CS&EP is because of the CEO’s career horizon. A newly appointed CEO has a longer anticipated career than those who are near retirement (Khan et al., 2020b). Therefore, CEOs in their early tenure are more likely to invest in social and environmental projects, and this leads to them being rewarded in their later service tenure by enhancing future firm value. This is in keeping with Pan et al. (2016), who suggest that a CEO’s investment activities are significantly higher in their early service tenure. However, social practices also foster long-lasting advantages for firms. Therefore, we posit that CEOs are more focused on CS&EP in their initial service years than they are in their final service years. Taken together, we propose that CEO tenure has a negative effect on CS&EP. Based on these justifications, we propose the following hypothesis.

**H1.** CEO tenure has a negative and significant impact on CS&EP.

### Coastal Areas Effect

In this section, we strive to elaborate on how the effect of CEO tenure on CS&EP varies between coastal and non-coastal areas of China. In a recent study, Li et al. (2019) observed that increased social practices are taking place in the highly developed areas of China’s coastal zones. In addition, using data envelopment analysis, Wang et al. (2016) documented that the CSR efficiency score has a stable pattern in most provinces and in cities situated in the coastal areas of China. Our argument that social and environmental practices are attracting more attention in the coastal zones of China is based on the following.

First, coastal regions in China are more developed, and these populated areas contribute more than 55% of the country’s GDP (Wang, 1992). There are some social and environmental risks created by fast economic development that needed to be addressed as a matter of priority (Wang et al., 2016). Therefore, on 1st January 2006, the Chinese government—being a key stakeholder—implemented a new law forcing firms to be socially responsible in their operations (National People’s Congress [NPC], 2005). As per article-five of National People’s Congress law, companies are bound to engage in social practices and manifest their business ethics and social morality in a real way. Moreover, ICZM is overseen by the Chinese government. This is an internationally accepted management tool used to resolve conflicts regarding economic development, social responsibility, and pollution to protect the ongoing development of the country’s coastal zones (Chen and Pearson, 2015). Taken altogether, we postulate that CS&EP is more focused on the coastal areas of China, and increase in CEO tenure may increase CS&EP because CEOs’ career-related concerns remain constant throughout their careers due to excessive government pressure (Liu and Zhang, 2017).

**FIGURE 1:**
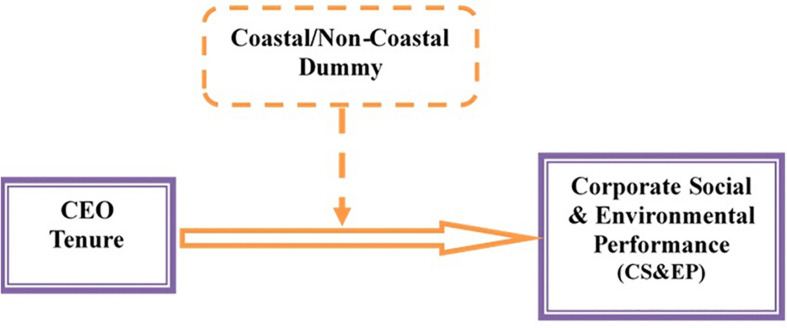
Moderating role of coastal/non-coastal areas.

Second, social performance is the basic requirement for Chinese firms to be able to compete internationally. Therefore, multinational corporations, like Carrefour, Nike, and GE, are strictly monitoring the CSR evaluation processes of their Chinese supplier firms (Ying et al., 2006), and almost 8,000 Chinese companies situated in the coastal zones are facing factory evaluations related to their social activities. Hence, one of the main tasks of Chinese firms is to upgrade their socially responsible competitiveness in the international market. Moreover, labor trade barriers and green trade barriers have pushed Chinese firms to be more focused on social activities in coastal zones. Therefore, we postulate that the negative relationship between CEO tenure and CS&EP is weaker in the coastal areas of China (Figure 1), which leads to the following hypothesis:

**H2.** The negative effect of CEO tenure on CS&EP is moderated by coastal areas. Specifically, this negative association becomes weaker in the firms with headquarters situated in the coastal areas of China.

### Value-Added Hypothesis

We argue that a CEO’s early service tenure engagement in CS&EP activities increases their compensation by mitigating career-related concerns. Extant research has shown that the CEO’s early service years’ engagement in social activities could be a helpful tool with which to reduce career-related concerns (Oh et al., 2014) and ultimately increase the CEO’s compensation (Berrone and Gomez-mejia, 2009). However, other studies have shown that the increase in social activities may reduce the CEO’s compensation (Coombs and Gilley, 2005; Russo and Harrison, 2005), meaning that the results are mixed. Therefore, we have extended this line of research in an emerging market to address this underlying issue.

The increase in social involvement increases shareholder wealth because the improved social investment is correlated with a high-quality workforce (Greening and Turban, 2000), increases demand for a firm’s product in the market (Navarro, 1988), leads to stronger customer loyalty to a firm’s products (Sen and Bhattacharya, 2001), and creates more convenience to attract quality resources (Cochran and Wood, 1984). On the other side, existing studies show that social practices are more likely to enhance a firm’s non-financial performance through better product quality (Johnson and Greening, 1999) and operational efficiencies (Sharma and Vredenburg, 1998). These studies align with stakeholder wealth maximization theory (Cornell and Shapiro, 1987; Hill and Jones, 1992), which suggests that firms act according to the contract between stakeholders (i.e., customers, employees, and suppliers) and shareholders. Each set of stakeholders provides firms with significant resources against the terms mentioned in the direct contract (i.e., product guarantee and wages contract) or indicated in indirect contracts (i.e., ongoing services to customers and promising job security to employees). If an increase in social investment is associated with stakeholders’ interests (i.e., customers, employees, and suppliers), these stakeholders will work to support the betterment of the firm’s operations, which ultimately enhances the shareholders’ wealth. Social practices are considered an important indicator to enhance shareholders’ wealth. Focusing more on securing the stakeholders’ interests ultimately makes them more inclined to support the firm’s operations, which results in an increase in the shareholders’ wealth. In this line of research, since in the initial service tenure of a CEO the gradual increase in social and environmental investment is associated with the betterment of the firm’s shareholders’ wealth, we anticipate that the CEO would be rewarded for their commitment to enhancing CS&EP, which would consequently reduce their career-related concerns. Therefore, we posit that there is a positive relationship between the CEO’s initial service CS&EP and total compensation.

**H3.** The increase in a CEO’s early service tenure CS&EP enhances their total compensation.

## Research Design

### Sample Selection

This study finds an association between CEO tenure and CS&EP in coastal and non-coastal areas of China. Our sample consists of 3,282 unbalanced observations of non-financial Chinese listed firms from 2009 to 2015. We retrieve our data from China Stock Market and Accounting Research (CSMAR), which incorporates firms’ financial and board composition data (Gao et al., 2017). Moreover, CS&EP data are extracted from the Rankins (RKS) dataset (Shahab et al., 2018; Sial et al., 2018).

### Measures

#### Corporate Social and Environmental Performance

Corporate social and environmental performance is a dependent variable in this study. The data are extracted from the RKS dataset available in the “HEXUN” database for Chinese listed firms. RKS is a private firm operating under the guidelines set by the Global Reporting Initiative. The extant literature has used the RKS dataset to conduct CS&EP research (Marquis and Qian, 2013; Shahab et al., 2018; Sial et al., 2018). Moreover, Marquis and Qian (2013) have implemented some robustness techniques to assure validity of the measures in RKS dataset. The RKS dataset uses three leading indicators in their evaluation process, which are shown in Table 1. Each factor is rated from zero to 100. The total firm rating quality score is measured by incorporating the weighted average of all three indicators from zero to 100.

**TABLE 1:** Three indicators from the Rankins (RKS) dataset.

Factors	Explanation
Technical prediction	Technical prediction consists of the regularity and availability of environmental/social statistics and transparency concerns.
Content prediction	Content prediction includes the acquiring of positively involved management and a framework for efficiently implementing economic social and environmental practices.
Overall prediction	The overall prediction indicator includes the social procedures and practices of the firm, the modernization of CSR activities, and the engagement of all stakeholders in social practices, along with the comparison of CSR disclosure reports.

#### CEO Compensation

We retrieve CEO compensation data from CSMAR. These data are based on the reported sums of CEO pay, stipends, and bonuses, consistent with Conyon and He (2012).

#### CEO Tenure

CEO tenure is the independent variable in this study, which is measured by the total number of years an executive has held the chief executive office (Hu et al., 2015; Chen and Tsang, 2017).

#### Coastal Dummy

**FIGURE 2:**
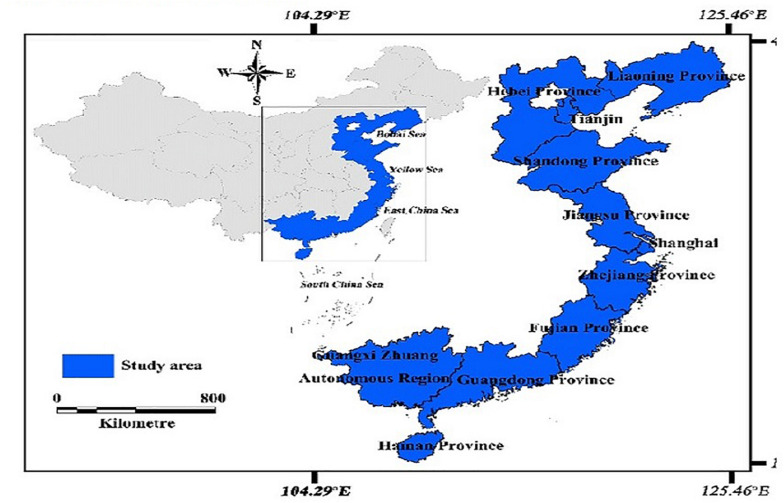
Coastal areas of China.

We segregate our sample with the help of the Chinese coastal area map provided in Figure 2 (Sun et al., 2015). We use a coastal zone dummy variable which equals one if the firm’s headquarters is situated in the coastal region of China and zero if not (Fleisher and Chen, 1997; Purdy and Chang, 2014).

### Model Specification

We propose the following models for this study.

$$\text{CS}\&{\text{EP}}_{i,t}=\alpha +\beta_{1}\,\mathrm{CEO}\,{\mathrm{TNUR}}_{i,t-1}+\beta_{2}$$

$$\mathrm{CEO}\,\mathrm{Level}\,\_\,{\text{Controls}}_{i,t-1}+\beta_{3}$$

(1) $$\mathrm{Firm}\,\mathrm{Level}\,\_\,{\text{Controls}}_{i,t-1}+\mu_{i,t}$$

$$\text{CS}\&{\text{EP}}_{i,t}=\alpha +\beta_{1}$$

$$\mathrm{CEO}\,\mathrm{TNUR}\times {\mathrm{COASTALDMY}}_{i,t-1}+\beta_{2}$$

$$\mathrm{CEO}\,\mathrm{Level}\,\_\,{\text{Controls}}_{i,t-1}+\beta_{3}$$

(2) $$\mathrm{Firm}\,\mathrm{Level}\,\_\,{\text{Controls}}_{i,t-1}+\mu_{i,t}$$

$$C\,E\,O\,\_\,C\,O\,M\,P_{i,t}=\alpha +\beta_{1}$$

$$C\,S\&E\,P\,{\left(I\,n\,i\,t\,i\,a\,l\,s\,e\,r\,v\,i\,c\,e\,y\,e\,a\,r\,s\right)}_{i}+\beta_{2}$$

$$\text{ROA}\,{\left(\mathrm{initial}\,\mathrm{service}\,\mathrm{years}\right)}_{i}+\beta_{3}$$

$$\mathrm{CEO}\,\mathrm{Level}\,\_\,{\text{Controls}}_{i}+\beta_{4}$$

(3) $$\mathrm{Firm}\,\mathrm{Level}\,\_\,{\text{Controls}}_{i}+\mu_{i,t}$$

In model 1, CS&EP is a dependent variable and CEO tenure (CEO TNUR) is taken as an independent variable. To investigate the association between CEO tenure and CS&EP, we control for a few aspects that have been mentioned in the existing literature. Specifically, we control for CEO age (CEO AG), assuming that younger CEOs have more opportunities to become involved in social activities than older CEOs (Oh et al., 2014; Khan et al., 2020a). We include CEO duality (CEO DLTY) as a control variable and anticipate an inverse impact of CEO duality on social activities, because CEO duality and corporate social reputation have a negative relationship (Lu et al., 2015). CEO gender (CEO GNDR) is the most frequently considered difference in the related research. Still, the findings are diverse. Zhuang et al. (2018) investigated and found that a higher number of female executives decreases the rating standard of the corporation. Moreover, related research shows that a higher percentage of female executives has a positive impact on social performance (Manner, 2010). CEO education (CEO EDUC) has a significant relationship with the firm’s social performance (Manner, 2010); therefore, we control for CEO education to examine its impact on CS&EP.

Some governance control variables are also used. We control the percentage of independent directors observing firm performance. Independent boards are accountable for protecting the shareholders’ interests, whereas organizations with an extended number of independent boards more actively execute social practices (Ortas et al., 2017). The extant literature documents a positive and significant association between ownership concentration (OWNR CONCN) and social practices (Wang et al., 2013; Lahouel et al., 2014), while previous literature further evaluates and finds a negative impact of ownership concentration on social practices (Dam and Scholtens, 2013).

We further include several firm-level control variables. We anticipate that older (F. AGE) and larger (F. SIZE) organizations are more likely to be involved in social practices because they have more investment resources (McGuinness et al., 2017; Shahab et al., 2018; Sarfraz et al., 2020a). Therefore, we expect firm age and firm size to have a positive impact on CS&EP. In addition, the firms that depend more on leverage ratios are less interested in social projects. The extant research has found that more leveraged firms tend to decrease their corporate social performance (Shahab et al., 2018; Khan et al., 2019). Following this argument, we expect to find an inverse association between a firm’s leverage and its CS&EP.

In model 2, we introduce an interaction term of CEO tenure with a coastal dummy (COASTAL DMY) to find the moderating effect of coastal area firms on the association between CEO tenure and CS&EP. Moreover, in model 3, we use CEO compensation (CEO COMP) as a dependent variable to assess the impact of a CEO’s CS&EP in their initial service years (initial service years) on their compensation package, which is equivalent to the average CS&EP of the initial 4 years of the CEO’s service period. We also use firm performance measure return on asset ROA (initial service years) as a control variable. Firm performance is a significant judgment criterion that cannot be ignored when assessing an executive’s compensation contract. The rest of the control variables in model 1 are also used in the evaluation process.

## Main Results and Analyses

### Descriptive Analysis of the Variables

The present study uses Stata software to analyze the data. Stata is a multipurpose program able to produce statistical analyses, simulations, data management, graphics, and regression analyses. The descriptive summary of all the important variables is presented in Table 2. CS&EP is a dependent variable in this study, having mean and median values of 38.45 and 35.08, respectively. The standard deviation value of CS&EP is 13.27, suggesting a notable distinction in CS&EP between the sample firms. CEO tenure (CEO TNUR) is an independent variable having mean and median values of 6.02 and 5, respectively.

**TABLE 2:** Descriptive analysis.

	No. of observation	MEAN	SD	Quarter 1	Median	Quarter 2
CS&EP*_*t*_*	3,282	38.450	13.271	29.548	35.089	43.807
CEO TNUR*_*t*_*_–__1_	3,251	6.022	3.626	3.000	5.000	9.000
LCTNUR*_*t*_*_–__1_	3,282	0.273	0.445	0.000	0.000	1.000
CEO AG*_*t*_*_–__1_	3,251	53.348	6.395	49.000	53.000	58.000
CEO DLTY*_*t*_*_–__1_	3,282	0.167	0.373	0.000	0.000	0.000
CEO GNDR*_*t*_*_–__1_	3,251	0.967	0.178	1.000	1.000	1.000
CEO EDUC*_*t*_*_–__1_	2,535	3.677	0.874	3.000	4.000	4.000
P. INDBRD*_*t*_*_–__1_	3,278	45.141	14.454	33.333	40.000	50.000
F. SIZE*_*t*_*_–__1_	3,282	23.263	1.788	22.020	22.997	24.142
F. AG*_*t*_*_–__1_	3,282	16.543	5.094	13.000	16.000	20.000
OWNR CONCN*_*t*_*_–__1_	3,282	59.240	17.067	47.630	59.340	71.380
LEVG*_*t*_*_–__1_	3,282	0.519	0.213	0.361	0.532	0.675
ROA*_*t*_*_–__1_	3,065	6.8300	6.542	3.412	5.932	9.511
COASTAL DMY*_*t*_*_–__1_	3,271	0.561	0.496	0.000	1.000	1.000

*See section “Appendix 1” for variable details.*

Table 2 also exhibits the mean and median values of CEO age (CEO AG) as 53.34 and 53 years, indicating that the average age of a CEO in a Chinese firm is 53 years. The mean value of CEO duality is 0.16, meaning that 16.7% of CEOs also hold a chair position in the firm’s board. The average value of CEO gender (CEO GNDR) is 0.96, indicating that 96% of our selected sample is male. Approximately 45% of the directors in our sample are independent (P. INDBRD). The average value of firm size (F. SIZE) is 23.26. Moreover, the mean and median values of firm age (F. AG) are 16.54 and 16 years, respectively. The mean and median values of ownership concentration (OWNR CONCN) are 59.24 and 59.340, indicating that Chinese organizations are more concentrated and monitored by the top ten shareholders. The average value of leverage (LEVG) is 0.51. The selected sample firms are profitable (ROA mean value = 6.83), and approximately 56% of the sample firms’ headquarters are situated in coastal areas of China (see section “Appendix 1” for an explanation of the variables.).

The correlation matrix is presented in Table 3. The correlation parameter between CS&EP and CEO TNUR is -0.02. The value is less than the significance level of 1%, supporting hypothesis H1. Table 3 shows that multicollinearity does not exist among the selected variables. Moreover, we tested the variance inflation factors (VIF) to examine the multicollinearity issue in the given dataset. The VIF value is 3.24, indicating that multicollinearity is not an issue in the given dataset because the VIF value is less than five (Baccouche et al., 2013).

**TABLE 3:** Pearson correlation matrix (*N* = 3282).

	01	02	03	04	05	06	07	08	09	10	11	12	13	14
CS&EP*_*t*_*	1.000													
CEO TNUR*_*t*_*_–__1_	−0.026^*s*^	1.000												
LCTNUR*_*t*_*_–__1_	−0.047^*s*^	0.849^*s*^	1.000^*s*^											
CEO AG*_*t*_*_–__1_	0.077^*s*^	0.151^*s*^	0.155^*s*^	1.000										
CEO DLTY*_*t*_*_–__1_	−0.030^*s*^	0.060^*s*^	0.095^*s*^	−0.126^*s*^	1.000									
CEO GNDR*_*t*_*_–__1_	0.036	0.022^*s*^	0.008^*s*^	0.119^*s*^	−0.000^*s*^	1.000								
CEO EDUC*_*t*_*_–__1_	0.154^*s*^	−0.089^*s*^	−0.085^*s*^	−0.236^*s*^	0.058^*s*^	0.031^*s*^	1.000							
P. INDBRD*_*t*_*_–__1_	0.111^*s*^	0.001^*s*^	0.008^*s*^	0.037^*s*^	0.106^*s*^	−0.006^*s*^	0.012^*s*^	1.000						
F. SIZE*_*t*_*_–__1_	0.504^*s*^	−0.049^*s*^	−0.067^*s*^	0.221^*s*^	−0.102^*s*^	0.058^*s*^	0.121^*s*^	0.092^*s*^	1.000					
F. AG*_*t*_*_–__1_	−0.090^*s*^	0.098^*s*^	0.125^*s*^	0.076^*s*^	0.014^*s*^	−0.085^*s*^	−0.066^*s*^	−0.046^*s*^	−0.098^*s*^	1.000				
OWNR CONCN*_*t*_*_–__1_	0.320^*s*^	−0.187^*s*^	−0.202^*s*^	0.017^*s*^	−0.047^*s*^	0.036^*s*^	0.109^*s*^	0.072^*s*^	0.312^*s*^	0.334^*s*^	1.000			
LEVG*_*t*_*_–__1_	0.135^*s*^	−0.046^*s*^	−0.052^*s*^	0.069^*s*^	−0.105^*s*^	0.033^*s*^	0.067^*s*^	0.014^*s*^	0.531^*s*^	0.057^*s*^	0.003^*s*^	1.000		
ROA*_*t*_*_–__1_	−0.004^*s*^	0.067^*s*^	0.081^*s*^	0.036^*s*^	0.052^*s*^	−0.047^*s*^	−0.100^*s*^	−0.025^*s*^	−0.027^*s*^	−0.050^*s*^	0.132^*s*^	−0.367^*s*^	1.000	
COASTAL_DMY*_*t*_*_–__1_	−0.001^*s*^	0.064^*s*^	0.080^*s*^	0.035^*s*^	0.091^*s*^	−0.018^*s*^	−0.134^*s*^	−0.034^*s*^	−0.085^*s*^	0.239^*s*^	−0.053	−0.043	−0.008	1.000

*See section “Appendix 1” for variable details. ^*s*^Symbol manifests the 5% level of significance.*

### Main Results

Table 4 depicts the full-sample regression results to determine the impact of CEO tenure on CS&EP. We can see that the parameter of CEO TNUR is negative but significant at the 1% level (0.07), suggesting that CEO tenure has a negative and significant impact on the firm’s CS&EP. The regression findings show that firms’ CS&EP is more excessive in the early tenure of their CEOs than in their later tenure, which is consistent with hypothesis H1. The present study used a Hausman test to choose between a fixed or random effects model. Our test results show that the *p*-value is significant at the 1% level (307.82^∗∗∗^), suggesting that the fixed effects model is preferable. The rest of the coefficients, i.e., ownership concentration (OWNR CONCN), firm size (F. SIZE), and firm age (F. AG), are positive and significant, consistent with the extant literature (Wang et al., 2013; McGuinness et al., 2017; Shahab et al., 2018; Shah et al., 2019; Li et al., 2020).

**TABLE 4:** The effect of tenure on CS&EP.

Dependent variable = CS&EP*_*t*_*	(a)		(b)	
	Parameter estimate	t. static	Parameter estimate	t. static
CEO TNUR*_*t–*_*_1_	−0.076**	−2.02		
LCTNUR*_*t*_*_–__1_			−0.608**	−2.01
CEO AG*_*t*_*_–__1_	−0.0376	−1.07	−0.043	−1.25
CEO DLTY*_*t*_*_–__1_	0.018	0.04	0.028	0.06
CEO GNDR*_*t*_*_–__1_	0.511	0.40	0.557	0.43
P. INDBRD*_*t*_*_–__1_	−0.003	−0.47	−0.003	−0.42
F. SIZE*_*t*_*_–__1_	1.469***	2.71	1.452***	2.68
F. AG*_*t*_*_–__1_	2.054***	19.79	2.041***	20.07
OWNR CONCN*_*t*_*_–__1_	0.038*	1.84	0.038*	1.85
LEVG*_*t*_*_–__1_	−2.502	−1.54	−2.441	−1.50
Constant	−25.935**	−2.30	−25.432**	−2.25
Hausman check	307.82***		319.19***	
No of observations	2,514		2,514	
R. Square	0.384		0.384	

See section “Appendix 1” for variable details. *, **, and *** is equal to significant level at <10, 5, and 1%, respectively.

In this study, CEO tenure is our key variable of interest, defined as the entire number of service years an executive carries the chief executive title. However, we anticipate that tenure might not affect the firm’s social practices monotonically. Thus, we modify model 1 in this study by adding longer-tenured CEOs LCTNUR rather than CEO TNUR. LCTNUR is a dummy variable that is equal to one if the tenure exceeds 10 years and zero otherwise (Chen and Tsang, 2017). Table 4, column (b) shows that the coefficient of LCTNUR is negative and significant (-0.60), indicating that the firm’s CS&EP decreases during the later tenure.

To further clarify the findings, we reexamine model 1 by replacing CEO TNUR with indicator variables for the initial 5 years of executive tenure (Ali and Zhang, 2015). We take Y. ONE, Y. TWO, Y. THREE, Y. FOUR, and Y. FIVE to equal one if the observation belongs to the initial 5 years of executive tenure and zero otherwise. Table 5 shows that the parameters of Y. TWO and Y. FOUR are positive and significant, having values of 0.82 and 0.93, respectively. This suggests that CS&EP increases in the second and fourth years of an executive’s tenure. The parameter estimate belonging to Y. THREE is marginally significant and positive (0.43). The parameter of Y. ONE is insignificant but also positive. The extant research has documented that executives are not effective in engaging social practices in their first year of service (Chin et al., 2013). Here, we can note that the parameter of Y. FIVE is insignificant and negative. Collectively, our results show that the CS&EP of firms enhances in the second, third, and fourth years of a CEO’s tenure; subsequently, the performance shows a decreasing trend in their later years of service.

**TABLE 5:** The effect of the CEO’s initial service years on CS&EP.

Dependent variable = CS&EP*_*t*_*	Parameter estimate	t. static
Y. ONE*_*t*_*_–__1_	1.118	1.39
Y. TWO*_*t*_*_–__1_	0.828*	1.68
Y. THREE*_*t*_*_–__1_	0.438	1.61
Y. FOUR*_*t*_*_–__1_	0.937*	1.83
Y. FIVE*_*t*_*_–__1_	−0.742	−1.35
CEO DLTY*_*t*_*_–__1_	0.019	0.04
CEO GNDR*_*t*_*_–__1_	0.126	0.10
P. INDBRD*_*t*_*_–__1_	−0.004	−0.51
F. SIZE*_*t*_*_–__1_	−3.664**	−2.09
F. AG*_*t*_*_–__1_	2.437***	16.17
LEVG*_*t*_*_–__1_	−1.248	−0.82
Constant	11.414***	3.49
Hausman check	614.67***	
No of observations	2,514	
R. Square	0.383	

See section “Appendix 1” for variable details. *, **, and *** is equal to significant level at <10, 5, and 1%, respectively.

### Endogeneity Test Using Two-Stage Least Squares

We used the Two-Stage Least Squares (2SLS) technique to address the endogeneity problem. To control the impact of omitted variables, we placed an instrument variable (IV) in our model. While running the first-stage regression analysis, we took the industry average of CEO tenure in the previous year (TNUR. INDAVG) as an IV. The variable TNUR. INDAVG may affect the tenure of the CEO but did not correlate with CS&EP. In the second stage of the regression, we deploy the predicted CEO tenure (P. CEOTNUR) derived from the regression findings of the first stage. We examine the effect of predicted CEO tenure on CS&EP. The regression results are shown in Table 6. The parameter of P. CEOTNUR is significant with a negative value of -0.77, indicating that the effect of tenure on CS&EP holds after resolving the endogeneity problem with the 2SLS method.

**TABLE 6:** Resolving the endogeneity problem using 2SLS.

Dependent variable	(a)		(b)	
	1st. Stage		2nd. Stage	
	=CEO TNUR*_*t–*_*_1_		=CS&EP*_*t*_*	
	Parameter estimate	t. static	Parameter estimate	t. static
P. CEOTNUR			−0.772***	−7.03
TNUR. INDAVG*_*t–*_*_1_	0.980***	33.97		
CEO AG*_*t–*_*_1_	0.101***	11.41	−0.089***	−2.66
CEO DLTY*_*t–*_*_1_	0.269*	1.76	0.611	1.14
CEO GNDR*_*t–*_*_1_	0.215	0.68	0.748	0.62
P. INDBRD*_*t–*_*_1_	0.001	0.40	0.262*	1.70
F. SIZE*_*t–*_*_1_	0.119***	2.84	4.648***	28.58
F. AG*_*t–*_*_1_	−0.018	−1.53	0.051	1.12
OWNR CONCN*_*t–*_*_1_	−0.032***	−8.88	0.123***	8.72
LEVG*_*t–*_*_1_	−0.768**	−2.29	−7.409***	−5.76
Constant	−5.778***	−6.22	−74.344***	−21.14
No of observations	2,514		2,514	
R. Square	0.376		0.400	

See section “Appendix 1” for variable details. *, **, and *** is equal to significant level at <10, 5, and 1%, respectively.

### Coastal Areas Effect

Table 7 presents the findings of hypothesis H2. In model 2, column (a), we interact CEO TNUR with COASTAL DMY, where COASTAL DMY is a dummy variable equal to one if the firm’s headquarters is situated in a coastal area of China and zero otherwise. The coefficient of CEO TNUR^∗^COASTAL DMY is significantly positive (0.160), suggesting that the firms with head offices in the coastal areas of China weaken the inverse effect of CEO tenure on CS&EP, while the coefficients of CEO TNUR and COASTAL DMY are also positively significant, indicating that CS&EP is more focused in the coastal areas of China than non-coastal ones. Our results further suggest that CEOs pay more attention to social and environmental investments in coastal areas due to the excessive pressure they face from various stakeholders. The results in column (b) are also consistent with the results in column (a). Our findings fill the gap in the limitations mentioned by Khan et al. (2020b) and provide evidence that CS&EP varies in coastal and non-coastal areas.

**TABLE 7:** CEO tenure and CS&EP: The impact of Coastal areas.

Dependent variable = CS&EP*_*t*_*	(a)		(b)	
	Parameter estimate	t. static	Parameter estimate	t. static
CEO TNUR*_*t–*_*_1_	0.305***	4.91		
LCTNUR*_*t–*_*_1_			1.804***	3.40
CEO TNUR*COASTAL DMY*_*t–*_*_1_	0.160**	2.02		
LCTNUR*COASTAL DMY*_*t–*_*_1_			1.678**	2.46
COASTAL DMY*_*t–*_*_1_	14.166***	2.59	14.587***	2.66
CEO AG*_*t–*_*_1_	0.170***	3.33	0.225***	4.51
CEO DLTY*_*t–*_*_1_	1.540**	2.28	1.399**	2.11
CEO GNDR*_*t–*_*_1_	−8.473***	−3.58	−9.083***	−3.82
CEO EDUC*_*t–*_*_1_	0.838**	1.95	0.999**	2.32
P. INDBRD*_*t–*_*_1_	0.0487***	4.84	0.050***	4.99
OWNR CONCN*_*t–*_*_1_	−0.067***	−2.37	−0.072***	−2.54
LEVG*_*t–*_*_1_	4.077**	1.97	4.247**	2.04
Constant	25.866***	4.95	24.513***	4.67
Hausman check	92,71***		90.46***	
No of observations	1,968		1,968	
R. Square	0.144		0.131	

See section “Appendix 1” for variable details. **, and *** is equal to significant level at <5, and 1%, respectively.

### Value-Added Hypothesis

Table 8 shows the results of model 3. We asked an interesting question that had not been addressed in the extant research regarding whether CEOs could be rewarded for their social and environmental commitment in their initial service years. The parameter of CS&EP is positively significant (0.25), indicating that the increase in a CEO’s CS&EP in their early tenure increases their total compensation, which is consistent with hypothesis H3. In other words, higher CS&EP is associated with improved shareholder wealth; therefore, rewarding CEOs with compensation packages ultimately reduces their career-related concerns. Our results align with Jian and Lee’s (2015) value-creation hypothesis, indicating that the increase in CS&EP is positively linked with CEO compensation.

**TABLE 8:** The impact of CS&EP on CEO Compensation.

Dependent variable = CEO. COMP*_*t*_*	Parameter estimate	t. static
CS&EP*_*t*_*	0.255*	1.67
ROA*_*t*_*	0.000	0.01
CEO AG*_*t*_*_–__1_	0.014*	1.84
CEO DLTY*_*t*_*_–__1_	0.063	0.53
CEO GNDR*_*t*_*_–__1_	−0.153	−0.52
P. INDBRD*_*t*_*_–__1_	−0.009 ***	−5.21
OWNR CONCN*_*t*_*_–__1_	−0.002	−0.43
LEVG*_*t*_*_–__1_	−0.880 ***	−2.38
Constant	11.812***	14.82
Hausman Check	29.32***	
No of observations	2,349	
R. Square	0.02	

See section “Appendix 1” for variable details. *, and *** is equal to significant level at <10, and 1%, respectively.

## Concluding Remarks

Due to the growth in stakeholders’ expectations and institutional changes, China—as the world’s second largest emerging economy—provides an attractive market for research. It enables us to investigate how CEO tenure influences firms’ CS&EP in coastal and non-coastal areas of China. The growing interest of scholars in the field of social and environmental practices in the Chinese context means that many studies have examined the association between executive attributes and firms’ economic, social, and environmental performance (Lu et al., 2015; McGuinness et al., 2017; Shahab et al., 2018). By extending beyond this line of research, we filled a gap by empirically scrutinizing the effects of CEO tenure on CS&EP in coastal and non-coastal areas of China. Moreover, we have contributed to the existing literature by providing evidence that a CEO’s CS&EP during their initial tenure may increase their compensation package. We used data of listed firms for the period of 2009 to 2015 and showed that CEOs with shorter service tenures (i.e., CEOs in their initial service years) are more likely to be involved in CS&EP than CEOs with longer service tenures. Our findings also demonstrate an interesting outcome, showing that CS&EP is more concentrated in the coastal areas of China. Our study provides evidence that the inverse effect of CEO tenure on CS&EP is weaker in coastal areas. Finally, we show that CEOs’ CS&EP in their initial service years increases their compensation packages by reducing their career-related concerns.

The present study has several implications for business leaders and policymakers as they develop strategies to make organizations more responsible for their social and environmental goals. Many researchers have documented a significant association between social practices and firm financial performance. Therefore, all the monitoring authorities (like governments and boards of directors) have considered the importance of social and environmental practices. Therefore, CEOs should be urged to focus more on CS&EP. Accordingly, many firms have taken serious steps to factor CS&EP into their assessments of CEO performance. In addition, our study provides evidence that CEO tenure is an essential determinant that could influence CS&EP. Moreover, governments and firms’ boards should set up CEO tenure-related benefits (i.e., increase CEOs’ compensation packages) to encourage CEOs to engage in social and environmental practices that could be equally beneficial for both shareholders and stakeholders. Executives’ policies regarding employee protection could also be helpful in reducing the spread of COVID-19 by implementing the guidance (i.e., social distancing) provided by the World Health Organization. Moreover, this study is also useful for the stakeholders involved in CS&EP evaluation and sustainable development, as well as mutual fund managers, to understand regional differences. By using our findings, these stakeholders can draw meaningful conclusions.

The present study could be extended to future research. We chose a sample of firms listed on the Shenzhen and Shanghai stock markets to explore the benefits related to social and environmental performance; thus, future studies could extend this line of research by investigating the impact of CEO tenure on social, economic, and environmental disclosure practices. Second, we recommend examining the moderating role of institutional investors and financial analysts on the link between CEO tenure and CS&EP. Third, since we used a sample of Chinese listed firms, our results may not be generalized to different territories. Future research could improve on this study by investigating how CEOs use social activities to signal their capabilities in different scenarios.

## Data Availability Statement

The raw data supporting the conclusions of this article will be made available by the authors, without undue reservation.

## Author Contributions

TK has contributed to the definition of research objectives, developing models, hypotheses, data analysis plan, article writing, revision and proofreading, and final approval. GB has contributed by developing models, hypothesis revision, and proofreading. ZF has contributed in research objectives, data analysis, and results’ interpretation. SQ has contributed in drafting the work, validation of results, and revision. ZK and WK have contributed to data collection, analysis, drawing limitations, future directions, and the conclusion of the study. All authors contributed to the article and approved the submitted version.

## Conflict of Interest

The authors declare that the research was conducted in the absence of any commercial or financial relationships that could be construed as a potential conflict of interest.

## Footnotes

1 http://www.csrhub.com/blog/2013/05/top-companies-tie-compensation-to sustainability.html

## Appendix

**APPENDIX 1:** Detail explanation of variables.

Symbol	Detail
**D.V:**	
CS&EP*_*t*_*	Corporate social and environmental performance is predicted by the Rankins dataset ratings from 1 to 100, for a particular firm in a certain year.
**I.V:**	
CEO AG*_*t*__–_*_1_	CEO age is defined as the age of the firm’s CEO in the previous year.
CEO DLTY*_*t*__–_*_1_	CEO duality is an Indicator variable = one if the firm CEO has a dual role, such as CEO and the board chairmanship in a previous year, and zero otherwise.
CEO GNDR*_*t*__–_*_1_ CEO EDUC*_*t*_*_–__1_	CEO gender is an indicator variable that is equal to one if the firm CEO is a male in a year of T-1, and zero otherwise. CEO education is an indicator variable = one if the CEO has a primary education, 2 for secondary education, 3 for master’s degree holders, and 4 for Ph.D. degree holders in the previous year, and zero otherwise
P. INDBRD*_*t*__–_*_1_	The percentage of an independent board of directors is predicted by calculating the% of the firm independent board in the previous year.
OWNR CONCN*_*t*__–_*_1_	Firm ownership-concentration is calculated by taking the sum of the ratio of all the shares owned by the ten large shareholders in the previous year.
F. SIZE*_*t*__–_*_1_	Firm size is calculated by proceeding the natural logarithm of the total assets of a firm in the previous year.
F. AG*_*t*__–_*_1_	The age of a firm is determined as the listing age of the firm in the previous year.
ROA*_*t*__–_*_1_	Firm return on asset is calculated by the net income divided by total assets in the previous year.
COASTAL DMY*_*t*__–_*_1_	Coastal dummy is an indicator variable which is equal to one if a firm headquarter is situated in coastal areas of China in a previous year, and zero otherwise.
LEVG*_*t*__–_*_1_	The leverage of a firm in a previous year is calculated by dividing total debts on total assets.
Y. ONE*_*t*__–_*_1_	Year one is an indicator variable which is equal to one if the observation pertains to the first service year of CEO tenure, and zero otherwise.
Y. TWO*_*t*__–_*_1_	Year two is an indicator variable which is equal to two if the observation pertains to the second service year of CEO tenure, and zero otherwise.
Y. THREE*_*t*__–_*_1_	Year three is an indicator variable which is equal to three if the observation pertains to the third service year of CEO tenure, and zero otherwise.
Y. FOUR*_*t*__–_*_1_	Year four is an indicator variable which is equal to four if the observation pertains to the fourth service year of CEO tenure, and zero otherwise.
Y. FIVE*_*t*__–_*_1_	Year five is an indicator variable which is equal to five if the observation pertains to the fifth service year of CEO tenure, and zero otherwise.
